# Using Nanomaterials as Excellent Immobilisation Layer for Biosensor Design

**DOI:** 10.3390/bios13020192

**Published:** 2023-01-27

**Authors:** Azeez Olayiwola Idris, Seyi Philemon Akanji, Benjamin O. Orimolade, Foluke Omobola Grace Olorundare, Shohreh Azizi, Bhekie Mamba, Malik Maaza

**Affiliations:** 1 UNESCO-UNISA Africa Chair in Nanoscience and Nanotechnology College of Graduates Studies, University of South Africa, Pretoria 392, South Africa; 2 Nanosciences African Network (NANOAFNET), iThemba LABS-National Research Foundation, Somerset West 7129, South Africa; 3 Petroleum Engineering, School of Engineering Department, Edith Cowan University, 270 Joondalup Drive, Perth, WA 6027, Australia; 4 Institute for Nanotechnology and Water Sustainability (iNanoWS), College of Science, Engineering and Technology, University of South Africa, Private Bag X6, Florida Science Campus, Johannesburg 1709, South Africa; 5 Department of Chemical Sciences, University of Johannesburg, Doornfontein, Johannesburg 2028, South Africa

**Keywords:** nanomaterial, biosensor, immobilisation, graphene, carbon nanotubes, carbon nanoparticles, carbon nanodots, MXenes

## Abstract

The endless development in nanotechnology has introduced new vitality in device fabrication including biosensor design for biomedical applications. With outstanding features like suitable biocompatibility, good electrical and thermal conductivity, wide surface area and catalytic activity, nanomaterials have been considered excellent and promising immobilisation candidates for the development of high-impact biosensors after they emerged. Owing to these reasons, the present review deals with the efficient use of nanomaterials as immobilisation candidates for biosensor fabrication. These include the implementation of carbon nanomaterials—graphene and its derivatives, carbon nanotubes, carbon nanoparticles, carbon nanodots—and MXenes, likewise their synergistic impact when merged with metal oxide nanomaterials. Furthermore, we also discuss the origin of the synthesis of some nanomaterials, the challenges associated with the use of those nanomaterials and the chemistry behind their incorporation with other materials for biosensor design. The last section covers the prospects for the development and application of the highlighted nanomaterials.

## 1. Introduction

The progress in the field of material chemistry and nanotechnology in recent times has paved the way for remarkable accomplishments in development. This progress is seen in the advent of novel materials and their hybrids, which now offer expectations as excellent immobilisation layers for biosensor development, suitable for onsite results delivery of a range of analytes (at a reduced amount) concurrently in the absence of specialised staff or complex device [1]. Briefly, biosensors are diagnostic devices that transform biological responses into electrical signals [2]. They routinely determine the number of biological markers or certain chemical reactions by producing the signals that are primarily associated with the analyte concentration present in the chemical reaction [2]. This routine determination is made possible through the aid of a bioreceptor, the biological sensing component of the biosensor usually connected to the transducer. The bioreceptor detects the analyte while the transducer transforms the recognisable response into a quantifiable signal. Because of the type of bioreceptors, biosensors can be grouped into enzymatic biosensors, immunobiosensors and genobiosensors. Further classification of biosensors based on the transduction process includes electrochemical, thermometric, piezoelectric and optical biosensors. Electrochemical biosensors, among other types of biosensors, are increasingly common, effectively marketed, and are numerous [3]. Further subdivision of electrochemical biosensors includes potentiometric, amperometric and impedimetric biosensors [1]. 

Given the fact that Clark and Lyons were the first to utilise an oxygen electrode as a glucose biosensor in 1962 [4,5], numerous conductive electrodes including gold (Au), platinum (Pt), silver (Ag), glassy carbon, screen-printed among others have been exploited for biosensor construction. Nevertheless, the performance of biosensors is often restricted using bare electrodes. Therefore, utilising nanomaterials as an immobilisation layer is critical to increase the surface area of an electrode for better biosensing performance. As a result, electrodes modified with nanomaterials not only improve the immobilisation of functional recognition molecules, but also reduce nonspecific binding as well as augment the transfer of electrons in the process of catalytic reaction. In light of the outstanding developments in material chemistry and nanotechnology, numerous nanomaterials have been developed through different synthetic approaches [5]. The European Commission (EU) (2017), defined nanomaterials as natural, incidental or synthetic material with particles not less than one dimension in a nanosized scale between 1 and 100 nm, in a boundless state or as a combined or an agglomerate as well as where 50% or more of the particles are in the nanosized grouping [6]. The nanosized dimension provides exceptional qualities to nanomaterials such as a high surface area to volume ratio, causing diverse exceptional physical and chemical attributes like increased catalytic impact, mechanically robust, high molecular adsorption, and immense surface tension driven together with prolonged chemical and biological activities [7]. Carbon-based nanomaterials (like graphene, and carbon nanotubes), MXenes, nanowires, nanosheets, nanoflowers dendrimers, quantum dots (QDs), metal and metal oxide nanomaterials as well as their nanocomposites have been exploited as excellent immobilisation layers for the design of novel biosensors in areas such as biomedical and human healthcare field [1,5,8]. To mention a few, a review highlighting the recent advances in superparamagnetic nanostructures like Fe_3_O_4_ and Fe_2_O_3_ for electrochemical and optical biosensing for disease-specific biomarkers was reported [8]. The utilisation of graphene, carbon nanotubes, zinc oxide and gold as top nanomaterials for inventions of biosensors for healthcare had also been reported [1]. The immobilisation of Pt–Pd bimetallic nanoparticles, dispersed in an ionic liquid and peroxidase on nanoclay in the development of a biosensor had also been reported [9]. 

Based on the aforementioned, this review focuses mainly on the application of nanomaterials carbon nanomaterials and underutilised MXenes as excellent immobilisation layers for biosensors design.

### 1.1. Carbon Nanomaterials

Carbon as a natural element has received increased attention for some time. The consistent advancement of nanotechnology has increased the understanding of carbon materials from macroscopic to nanoscale (10). Today, various allotropes of carbon nanomaterials ranging from zero-dimensional (0D) to three-dimensional (3D) have been applied in biosensing [10], energy storage and conversion [11], nanoelectronics and high-frequency electronics [12,13], field emission displays [14] and theranostics [15]. In general, the wide use of carbon materials as outstanding immobilisation materials in biosensing is due to a broad potential window and chemical inertness [10]. Among the varieties of carbon nanomaterials, the section below discusses the application of graphenes (including its derivatives), carbon nanotubes, carbon nanodots and carbon nanoparticles as immobilisation layers for biosensors development.

### 1.2. Graphene and Its Derivatives 

Graphene (GR) exists as a single-atom-thick planar sheet with sp^2^-bonded carbon atoms entirely arranged in a honeycomb lattice [10]. It is a two-dimensional (2D) carbon nanomaterial with stable and durable configuration, desirable resistance and flexibility properties, enhanced surface area (~2600 m^2^ g^−1^), lightweight, outstanding thermal conductivity and excellent electron transfer ability [11,16,17]. Using chemical oxidation, graphene can be transformed to graphene oxide (GO), which by further reaction can be transformed into its reduced form commonly known as reduced graphene oxide (RGO). The chemically transformed graphene materials possess several functional groups such as carboxyl, epoxide and hydroxyl units in contrast to pure graphene, thus imparting their easy dispersion ability in polar solvents, as well as an additional modification with biological molecules via covalent or non-covalent bonding [16,18]. In addition, the defects caused by the chemical transformation of graphenes endow them with diverse and advantageous processing properties [16,19,20].

Graphene is a fascinating choice as the matrix material for other nanomaterials in biosensor development. Functionalising graphene with diverse nanomaterials can further reveal synergistic effects through the use of their corresponding beneficial peculiarities [21]. For the record, Uzak and co-researchers described the construction of a novel glucose biosensor comprising nanocomposites of reduced graphene oxide (rGO), platinum nanoparticles (Pt NPs) and zinc-metal organic frameworks-74 (Zn-MOF-74) [22]. Generally, MOFs are crystalline porous and synthetic polymeric materials with highly organised structures made of the organic linker and metal ions that keep the framework undivided [23]. Structurally, the M-MOF-74 (M: Zn, Mg, Ni and Co) embodied an organic linker, namely 2, 5-dihydroxyterephthalic acid [22,24]. Nevertheless, rGO as a famous outstanding advanced-conducting material was fused with PtNPs and Zn-MOF-74 to produce hybrid nanomaterial for glucose oxidase (GOx) immobilisation owing to the weak electron conductivity of MOF-derived nanomaterials. The hybrid nanomaterials rGO/PtNPs/Zn-MOF-74 were synthesised as reported [22] and used as immobilisation layers for GOx to develop a novel glucose biosensor (GOx-rGO/Pt NP@Zn-MOF-74) using a glassy carbon electrode (GCE). The glucose biosensor design involves depositing rGO/Pt NPs on Zn-MOF-74 using of π–π interactions, while the favourable surface aided the immobilisation of GOx via the hydrogen bonds. The synthesised rGO/Pt NPs@Zn-MOF-74 hybrid nanomaterial suspension was used to modify the GCE by the drop-casting technique. Overall, the GOx-rGO/Pt NPs@Zn-MOF-74 biosensor demonstrated a range of linear measurement for glucose between 0.006–6 mM, with a limit of detection of 1.8 µM (S/N = 3) as well as a sensitivity of 64.51 µA mM^−1^ cm^−2^. Further, the amperometric response of the enzyme biosensor exhibited the usual behaviour of Michaelis–Menten kinetics with K_m_ value calculated as 2.21 mM. This lower value of K_m_ suggested a higher affinity of the enzyme towards its substrate. It also substantiated the fortification of the normal structure of GOx in rGO/Pt NPs@Zn-MOF-74 in addition to the rapid substrate diffusion. For real-time analysis, the acquired satisfying sensitivity and range of measurement aided fast and accurate glucose measurement in cherry juice via the developed biosensor [22]. Although the sensitivity of this sensor is impressive the author fails to discuss the chemistry of interaction between the nanomaterials used in the biosensor fabrication, and in our opinion the sensor is not affordable because the Pt used in the sensor development is expensive. 

Nowadays, breast cancer is known to be a global health concern [25]. It is also the leading cause of cancer in women [26]. Existing progress in molecular biology has revealed cancer biomarkers as a vital tool in diagnosis, prognosis and providing awareness into the causes of cancer [27]. In general, biomarkers are among the principal critical tools for early testing of cancer, grouping, staging, progression monitoring and assessment of immunisation against chemotherapy. Owing to this fact, the overall use of biomarkers in healthcare will eventually depend upon the discovery of many biomarkers with outstanding selectivity and sensitivity, such that they can discover even minor deviations in the amount of the markers in mixed biological fluids [27]. Based on this precedent, researchers recently reported the exploitation of 3D-reduced graphene oxide (3D-rGO) and polyaniline nanofibers to construct an electrochemical DNA biosensor using a GCE for highly sensitive detection of breast cancer BRCA1 biomarker [28]. To prevent more women from cancers arising from BRCA1 gene mutation, detection of BRCA1 gene mutation is of crucial importance as it usually facilitates diagnosis and intervention [29,30]. Recently, 3D-rGO has been reported to display excellent bioelectrochemical performance owing to its enhanced surface area, special morphology and structure, a lot of electroactive sites as well as the expedition of electron transfer [31,32]. PANI nanofibers, on the other hand, are an effective conductive electroactive polymer with large surface area, mechanical flexibility, adjustable conductivity, chemical uniqueness as well as simple processing, hence the suitability as a potential material in the development of extremely sensitive biosensors [33,34,35,36]. Additionally, graphene and PANI have similar conjugated π electrons structures, which gives them great superiority in the preparation of nanocomposites for the enhancement of electrochemical performance [37,38,39]. Owing to these reasons, the authors combined the excellent features of 3D-rGO and PANI nanofibers. This combination resulted in a tremendous improvement in the electrochemical activity of 3D-rGO-PANI/GCE, hence, a reduction in the charge transfer resistance (R_ct_) was observed using the electrochemical impedance spectroscopy (EIS) technique. This observation was reported to be due to the synergistic impact of 3D-rGO and PANI nanofibers. More so, the proposed biosensor was reported to successfully detect BRCA1 in actual blood samples with no obvious interference in the biosensor selectivity [28]. In our opinion, the author should have used an environmentally friendly reducing agent like ascorbic acid to reduce GO to graphene rather than using toxic hydrazine monohydrate as the reducing agent. 

The recent emergence of the photoelectrochemical (PEC) biosensor technique as an actively evolving technique for investigating different biological measures with a greater level of sensitivity is well documented [40]. PEC measurements, unlike electrochemistry, combine the merits of photochemistry and electrochemistry [41,42]. The fundamental of PEC biosensors is anchored on the photocatalytic oxidation or reduction in biological molecules to allow the movement of photogenerated electrons between the analyte as well as a semiconductor electrode under light emission to augment the PEC response. The advantage derived from the PEC technique led to a report on the development of a PEC immunosensing system involving phthalocyanine-sensitized graphene-cadmium sulphide (Pc-G-CdS) nanocomposites for prostate specific antigen (PSA) detection using ITO (Indium tin oxide) electrode [43]. CdS NPs were deposited on rGO which had been functionalised via non-covalent bonding, using sodium 1-pyrene sulfonate by taking the advantage of the π–π stacking communication between them. The light harvester—cobalt 2, 9, 16, 23-tetraaminophthalocyanine (CoTAPc)—was prepared on the G-CdS nanocomposites through electrostatic communication using self-assembly approach. According to the authors, the nanocomposites of CoTAPc-G-CdS displayed significantly higher and more stable photocurrent intensity in contrast to the nanocomposites of G-CdS, thus making the PEC biosensor system promising in the absence of a synchronised amplifier. Furthermore, a rise in the steric hindrance owing to immunocomplex formation was observed. This was evident in the photocurrent reduction over a PSA concentration range of 1 pg mL^−1^ to 5 µg mL^−1^. The observed detection limit was 0.63 pg mL^−1^. We recommend that the choice of materials used for photoelectrochemical biosensors must be photoactive, affordable and possess a large surface area. 

The grafting of water-soluble polymers containing reactive functional units to graphene or GO nanosheets was reported [44,45]. Arangue et al. explored this technique to design an electrochemical biosensor composed of a water-soluble reduced graphene oxide-carboxymethylcellulose hybrid nanomaterial for the design of a biosensor for catechol detection [46]. As a prototype, the authors employed the derived hybrid nanomaterial as a nanostructured framework for tyrosinase immobilisation on a glassy carbon electrode for biosensing of catechol. The employed hybrid nanomaterial was prepared via a reductive alkylation process involving two consecutive synthetic steps: (i) primary fortification of GO with amino units by grafting with (3-aminopropyl) triethoxysilane (APTES) moieties; and (ii) covalent link of periodate-activated CMC to GO. Thereafter, the graphene-based hybrid nanomaterial was employed as a layered material for GCE as well as frameworks for the carbodiimide-mediated covalent immobilisation of the enzyme tyrosinase. According to the authors, the morphological study of Tyr/rGO-CMC/GCE prompted the evaluation of the enzyme electrode for the fabrication of an amperometric biosensor for the detection of catechol. Remarkably, the tyrosinase biosensor displayed an outstanding analytical characteristic including a wide linear range of response between 20 nM and 56 µM, high sensitivity of 270 mA M^−1^ in addition to an extremely low detection limit of 0.2 nM for the amperometric determination of catechol. The successful outcome of the results obtained from this finding led the authors to conclude that the covalent link of anionic polysaccharides could be a successful means of developing water-soluble graphene-based hybrid nanomaterials for biosensors design. 

### 1.3. Carbon Nanotubes

**Figure 1:**
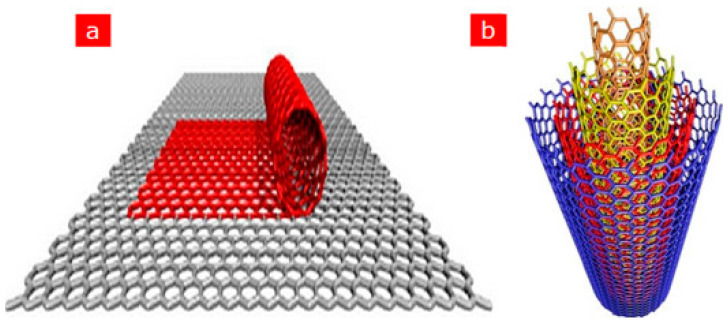
Schematic representation of (**a**) SWCNTs comprising a single graphite layer (**b**) MWCNTs comprising multiple graphite layers. Adapted from Ref. [47] with authorisation from Elsevier.

**Figure 2:**
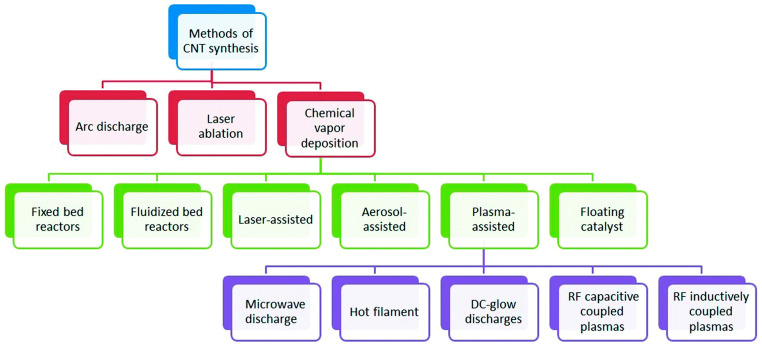
Categorization of synthesis methods used for carbon nanotubes. Adapted from Ref. [49] with Permission from RSC Advances.

After the first discovery of carbon nanotubes (CNTs) in 1991 by the Japanese scientist Iijima, CNTs have gained tremendous popularity as an immobilisation layer for biosensor design [7,47]. This prominence is because CNTs are electrically conductive with high tensile strength, a significant rate of Young’s modulus and outstanding catalytic features to several biological analytes in addition to their utilisation as a redox facilitator [48]. Structurally, CNTs are hollow cylindrical tubes composed of wrapped graphite sheets with a diameter ranging from nanometers to micrometers, made up of only sp^2^-hybridised carbon atoms. CNTs are classified into single-walled carbon nanotubes (SWCNTs) and multiwalled carbon nanotubes (MWCNTs). SWCNTs comprise a single layer of graphite sheet while MWCNTs comprise more than two layers of graphite sheet [12,47,49] (Figure 1 shows the schematic representation of SWCNTs and MWCNTs). Various synthetic approaches for CNTs include arc discharge, laser ablation and chemical vapour deposition (CVD) approach. While arc discharge and laser ablation procedures usually involve higher temperatures (>1700 ℃) for their synthesis, CVD, which has now replaced the latter procedures, is usually carried out at temperatures < 800 ℃ [49] (Figure 2 shows the summary of the categorisation of synthetic methods used for CNTs). Current studies revealed that CNTs hold outstanding electrochemical features owing to the existence of reactive units on their surface that can aid the electrons transfer of biomolecules. More so, improved electrocatalytic action of CNTs is associated with the existence of edge-plane-like locations situated toward the end as well as in the five-membered defects of CNTs. Overall, the voltammetric response of various biological molecules at CNT-modified electrodes usually presents higher peak currents as well as lower over-voltage. These outstanding features gave CNTs their tremendous popularity as an immobilisation layer for biosensor fabrication [7].

Furthermore, functionalisation of the physical and chemical features of nanoparticles is imperative to customise their use in electrochemical biosensors. This is often carried out by attaching some molecules on their surface. As a practical illustration, CNTs are insoluble in aqueous solutions; however, when they undergo oxidation in acidic mixtures the carboxylic units attached to the surface as well as the side walls of the nanotubes make them soluble in aqueous solutions. Hence, functionalisation has proven to be an effective means of altering the physical and chemical features of CNTs [50,51,52]. Notably, functionalisation of CNTs in surfactants containing solutions or with chemicals normally leads to a decrease in their bundle formation [53]. Covalent and non-covalent attachment are means by which CNTs can be functionalised with various chemical units and this makes them biologically compatible for conjugation with biomolecules, thus becoming promising immobilisation candidates for biosensor design [1,52]. Kumar et al. also added that amine and carboxyl functional units normally increase the electron transfer rate. In practice, they mentioned that water-based polymers built functionalisation of CNTs or surface functionalisation with ionic or hydrophilic units of CNTs usually aids CNT solubilisation in a water-based system. Based on their report, this was a substantial consideration for CNTs to act as a support medium for capturing biomolecules [1]. For example, an amperometric DNA biosensor based on GCE was reported for pathogens diagnosis [54]. The DNA biosensor was designed to primarily detect the exact IS6110 DNA sequence of *Mycobacterium tuberculosis* (MTB). Concisely, the authors employed AuNPs fused with functionalised fullerene (C60) to first create an appropriate biosensing platform to expedite transfer of electron and enhance the capture probe (CP). Thereafter, flower-like carbon nanotube-polyaniline (CNTs-PANI) nanohybrid were used to decorate the AuNPs to create a different tracker for the production and augmentation alert strategy, aided by an enzyme. Overall, the estimated limit of detection was 0.33 fM (S/N = 3) over a detection range of 1 fM–10 nM for the targeted DNA of MTB. The DNA biosensor was reported to demonstrate high specificity, sensitivity, and reproducibility for MTB detection in medical specimens. Although more medical specimens are needed to further demonstrate the practicability and competence of the proposed method in clinical use, it is a potent tool for early diagnosis of tuberculosis disease. In our opinion, the author was supposed to carry out a proof-of-concept experiment to identify the spectator nanomaterial and to confirm the role each modifier played in the biosensor development. 

Additionally, CNTs functionalisation increases direct electron transfer between the active sites of the bio-element and the electrode. Hence, redox polymers, hapten molecules, and thiol derivatives in addition to N-ethyl-N-(3-dimethylaminopropyl carbodiimide-N-hydroxyl succinimide (EDS-NHS) are predominantly used to functionalise MWCNTs [1]. For the record, the impact of CNT aids on the direct electron transfer (DET) as well as electrocatalytic activities of immobilised glucose oxidase (GOx) was studied by Yuxiang Liu and co-researchers [55]. GOx/CNT film was prepared by drop-casting a known amount of CNT suspensions and GOx solution was immobilised on (GCE) surface. Nafion was employed as a stabilizing solution to prevent the modifiers from leaking on the electrode surface during electrochemical analysis. Notably, a set of two distinct and basically reversible redox peaks were detected. According to the authors, this observation was an indication of a very good DET amidst the redox centres of GOx and GCEs in the absence of the electron transfer facilitators or metal NPs to connect with the flavin adenine dinucleotide (FAD) active centres of the GOx. Furthermore, the immobilised GOx by the CNTs preserved its electrocatalytic action against glucose. Notably, the authors emphasised that the DET and electrocatalytic action of GOx was heavily reliant on the amount of CNTs inner tubes. They further added that the impact of CNTs on the GOx electrocatalytic action coupled with the DET is probably because of the electron-tunneling impact over the outer wall and inner tubes of triple-walled CNTs. 

Bayram and Akyilmaz reported the development of a stable microbial biosensor based on Bacillus specie for the sensitive determination of paracetamol [56]. Notably, paracetamol (acetaminophen) is a commonly known analgesic and antipyretic that is used for treating pains. Its constant use is the reason why researchers studied the mechanism of its toxicity. The developed biosensor is composed of a gold electrode modified by carboxylated MWCNTs, polyaniline (PANI) with glutaraldehyde acting as a crosslinking agent. An amperometric technique was employed in the determination of paracetamol in drug samples at an applied potential of 0.5 V. The authors reported that the developed microbial system provided a significant cost-effective improvement for the detection of paracetamol with rapid and simple analytical method. Further, the π–π stacking communications between MWCNTs and PANI offered good stability and conductivity for the biosensor responses with an observed detection limit of 2.9 µM [56,57]. 

**Figure 3:**
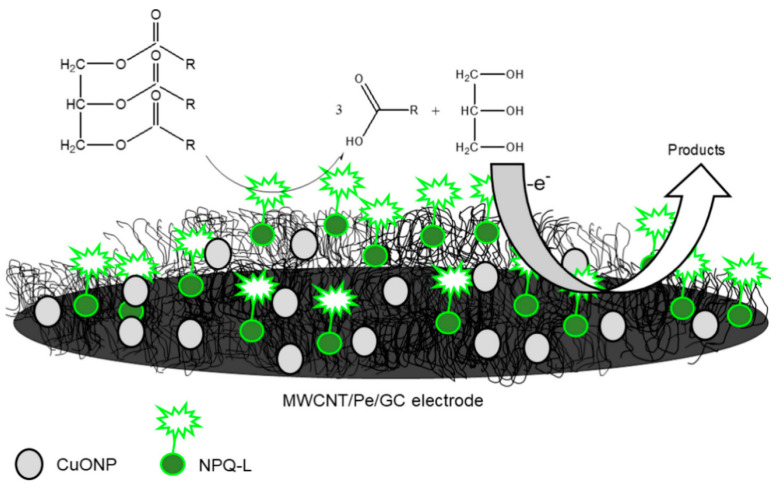
Graphic illustration of CNP-L/CuONP/MWCNT/Pe biosensor for determinations of triglycerides in serum specimens. Adapted from Ref. [66] with permission from Elsevier.

Furthermore, CNTs have been employed as supporting materials for both the dispersion and stabilisation of numerous organic and inorganic nanomaterials due to their extreme robustness coupled with massive reactive surfaces. The functionalisation of CNTs has steadily moved from organic to inorganic materials owing to the advancement of the chemistry of CNTs. Hence, CNTs are merged with diverse inorganic compounds like metal oxides, carbides, nitrides, chalcogenides as well as ceramics. Among the listed, metal oxides are considered the most commonly studied classes [7]. The integration of CNTs with metal NPs like Pt [58,59,60], Mo [61], Sn [62], Fe [63,64], and Cu [65] have been reported for several bioanalytical assays. For instance, Di Tocco and fellow researchers employed an electrochemical biosensor comprising immobilised lipase on chitosan-coated magnetic nanoparticles (CNP-L) on a multiwalled carbon nanotubes/pectin (MWCNT/Pe), modified with copper oxide nanoparticles (CuONP) using a GCE [66]. The biosensor was designed (Figure 3) to electrochemically detect total triglycerides (TGs)—commonly called natural fats—in serum samples. The detection of total glycerides is very important because it’s high levels, combined with cholesterol, are prominent causes of hypertension, coronary artery diseases and atherosclerosis [67]. The modification of the proposed biosensor at different stages—MWCNT/Pe/GCE, CuONP on MWCNT/Pe/GCE and CNP- L/CuONP/MWCNT/Pe/GCE—was carried out as reported [66,68]. Amperometry detection of TGs displayed a detection limit of 3.2 × 10^−3^–3.6 × 10^−3^ gL^−1^, quantification limits in the range 9.6 × 10^−3^–1.1 × 10^−2^ gL^−1^ in addition to a sensitivity of 1.64 × 10^−6^ AL g^−1^. The developed biosensor demonstrated an outstanding performance with 20 days of stability, good reproducibility, and repeatability. The developed analytical technique was also used for the investigation of TGs in standard human serum specimens. We recommend that when more than one modifier is used, the chemistry of interaction between the nanomaterials must be investigated and the modification route must be optimised.

### 1.4. Carbon Nanoparticles

Zero dimensional carbon nanoparticles (CNPs) [69], a popular carbon analogue—carbon nanotubes, graphene, and carbon dots [70]—possess special features such as biocompatibility, conductivity, non-toxicity and chemical inertness [71]. Hence, they are commonly utilised in biosensors as well as other nanotechnology platforms. CNPs hold similar sp^2^ electronic structure to graphene and CNDTs [69]. The ease of synthesis as well as cost-effectiveness are the merits of CNPs over other forms of carbon nanomaterials. For instance, the preparation of CNPs from candle soot is well documented [70,71]. For example, in our previous studies we combine the analytical merits of AuNPs and CNPs in the fabrication of an immunosensor for the detection of a cancer biomarker known as Alpha-fetoprotein (AFP) [70]. Concisely, the preparation of the immunosensor was carried out by immobilising anti-AFP probe (antibody) on a CNP/AuNPs nanocomposite-modified electrode for about 40 min at 35 ℃ accompanied by blockage against non-specific binding using bovine serum albumin (BSA). Overall, the combined impact of both nanomaterials resulted in a broad linear range of 0.005–1000 ng mL^−1^, detection limits by means of square wave voltammetry (SWV) of 0.0019 ng mL^−1^ and an EIS result of 0.00175 ng mL^−1^. In this study, a proof-of-concept study was carried out to identify the role each modifier played in biosensor development. It was observed that the CNP gave an improved current response from the gold nanoparticles, as revealed in the cyclic voltammetry and electrochemical impedance spectroscopy (EIS). Square wave voltammetry (SWV) and (EIS) were used to validate the detection limit obtained for AFP. 

A similar platform (CNPs-AuNPs) was employed to prepare aptasensors for arsenic (III) detection [72]. CNPs were employed as a signal booster, while AuNPs were utilised as an immobilisation layer for linking the aptamer to the electrode employing Au–S bond. The developed aptamer biosensor was not susceptible to any interference from Cd, Cu and Hg. It was reported that the developed biosensor was a substitute for alleviating interferences in arsenic detection. This aptasensor was constructed to resolve the popular interference effect of copper during arsenic sensing. 

**Figure 4:**
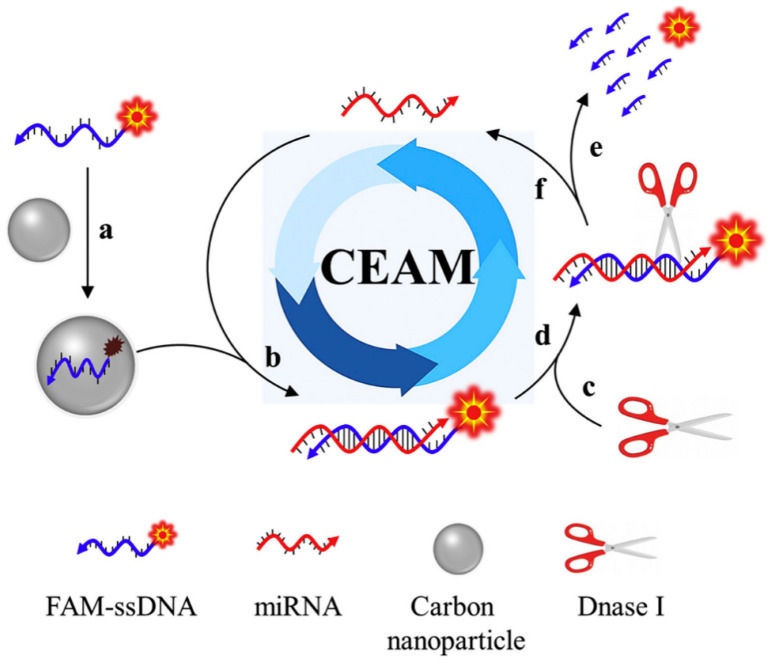
The operating principle of cyclic enzymatic amplification method (CEAM) for miRNA study. Primarily, FAM-tagged single-stranded DNA (FAM-ssDNA) coupled to the carbon nanoparticle surface (route **a**) give rise to a substantially quenched fluorescence signal. Formation of RNA/DNA hybrid (route **b**) upon introducing the target miRNA. The resultant attachment of DNase I (route **c**) identifies its suitable substrate: RNA/DNA hybrid (route **d**). Consequently, FAM-ssDNA is digested into a tiny portion and a strong fluorescence signal is produced (route **e**). Further, miRNA remains undisturbed and returns to its former status (route **f**), which is set up to initiate other reaction cycles; hybridisation, digestion, signaling and release. The recycling application of the target molecule provides an opportunity to successfully augment the detection sensitivity. Adapted from Ref. [75] with authorisation from Elsevier.

CNPs have been reported to display a good affinity with the target of interest when coupled with nucleic acid probes [73]. This is because CNPs usually demonstrate reversible as well as binding affinities over single-stranded nucleic acid probes, thus enabling CNPs to be used in nucleic acid-based sensing platforms [74]. Based on this foregoing, an intriguing study demonstrating a fluorescent biosensor based on CNPs and nucleic acid probes was reported. In this study, the authors employed the operating principle of the cyclic enzymatic amplification method (CEAM) for miRNA detection [75]. The study utilised nucleic acid probes for target discovery and signal augmentation, facilitated by DNase I. According to this study, an innovative blend of CNPs and nucleic acid probes is envisaged to unlock fresh ground in the advancement of smart analytical approaches that possess ample opportunities for clinical tests, environmental monitoring, and food security. The operating principle of the cyclic enzymatic amplification is shown in Figure 4.

### 1.5. Carbon Nanodots

Carbon nanodots (CNDTs) belong to quasi-spherical counterparts of carbon material with a size distribution below 10 nm. It is an amorphous nanocrystalline core which is sp^2^ hybridised in addition to an oxidised carbon surface comprising different functional units like hydroxyl, aldehyde and carboxyl units [76,77,78]. CNDTs remain an important part of the class of carbon nanomaterials owing to their exciting features like cost-effectiveness, outstanding biocompatibility, low toxicity, high aqueous solubility, photophysical features, strong chemical inertness, in addition to ease of synthesis [77,78,79,80]. For instance, the synthesis of CNDTs from naked oats has been reported [76,78]. Other methods of synthesis of CNDTs from different sources include arch-discharge, laser ablation, electrochemical, thermal routes, microwave-assisted, hydrothermal and aqueous-based techniques [81]. The exciting features possessed by CNDTs are the reason behind their use in several areas including sensing and biosensing [78,82,83,84], catalysis [85], drug delivery [86], bio-imaging [87], fuel cells [88] and dye-sensitisers [89]. However, there are still few reports available in the literature on the exploitation of CNDTs as an immobilisation layer for biosensor design. Furthermore, the synergistic impact of CNDTs when combined with other materials cannot be underestimated. To mention a few, a nano-mediator (polypropylene imine dendrimer (PPI) and CNDTs) was employed as a remarkable electron wire for the development of a biosensor for carcinoembryonic antigen (CEA) cancer biomarker on an underutilised exfoliated graphite electrode [78]. The idea of a synergic blend between PPI dendrimers as well as CNDTs was employed for CEA quantification. Additionally, the immobilisation of antibodies was aided by employing the host-guest supramolecular and biocompatibility features of dendrimers. The fabricated immunosensor gave rise to a low limit of detection of 0.00145 ng/mL over a linear range of 0.005–300 ng/mL. The developed immunosensor was finally applied for CEA determination in human serum specimens as proof of the potentiality of the immunosensor for real specimen analysis. The advantage of this sensor was that PPI was electrodeposited on the GCE electrode after CNP was drop-dried on the platform; this strategy helps to prevent the CNPs from leaking inside the electroanalytical solution. 

The application of poly (amidoamine) dendrimer (PAAD) functionalised CNDTs for extremely sensitive determination of alpha-fetoprotein (AFP) was reported [90]. Poly (amidoamine) dendrimer with rich amino units in their molecular backbones was employed as transporters for the immobilisation of additional carbon nanodots by employing covalent bond force [91]. Before immunoassay detection, a reduced graphene oxide (rGO) @fullerene (C_60_) modified electrode was first prepared and employed as a sensing medium to offer a wide surface area for covalent binding of capture antibody (Ab_1_) using a GCE. This step was followed by the self-assembling of detection antibodies (Ab_2_) via covalent binding on the PAADs@CNDTs composites, as sensitive electrochemiluminescent (ECL) bioprobes on the sensing interface for AFP detection using a simple sandwich immunoassay strategy. The strategy employed for AFP detection was based on the fact that as the ECL intensity of the fabricated immunosensor increases, the target antigen concentration also increases owing to the favourable electrochemical and ECL catalysis toward luminol [92]. This strategy gave rise to a low detection limit of 0.33 fg mL^−1^ over a broad active response of 1 fg mL^−1^–80 ng mL^−1^. The authors concluded that the PAADs functionalised CNDTs presented an optimistic method in clinical disease study [90]. 

**Figure 5:**
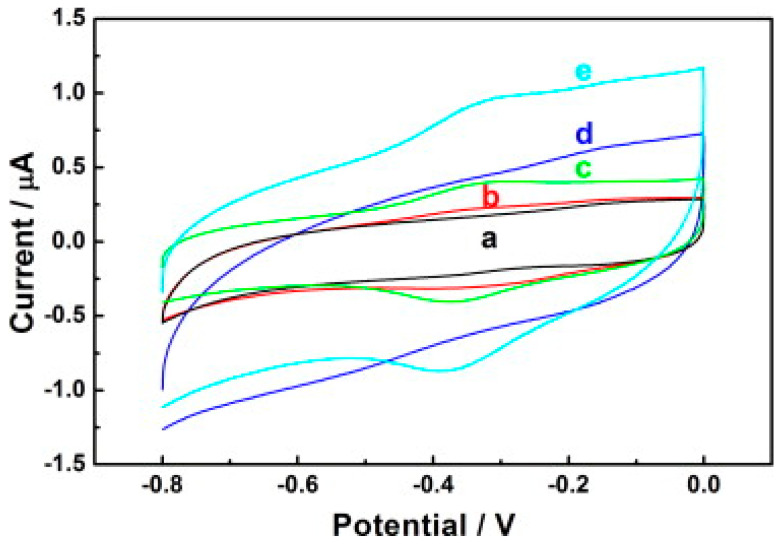
Cyclic voltammograms of (**a**) GCE (**b**) LDHs/GCE (**c**) HRP/LDHs/GCE (**d**) C-Dots/LDHs/GCE and (**e**) HRP/C-Dots/LDHs/GCE electrodes in 0.1 M PBS (pH = 7.0) at a scan rate of 50 mVs^−1^. Adapted from Ref. [93] with authorisation from Elsevier.

**Figure 6:**
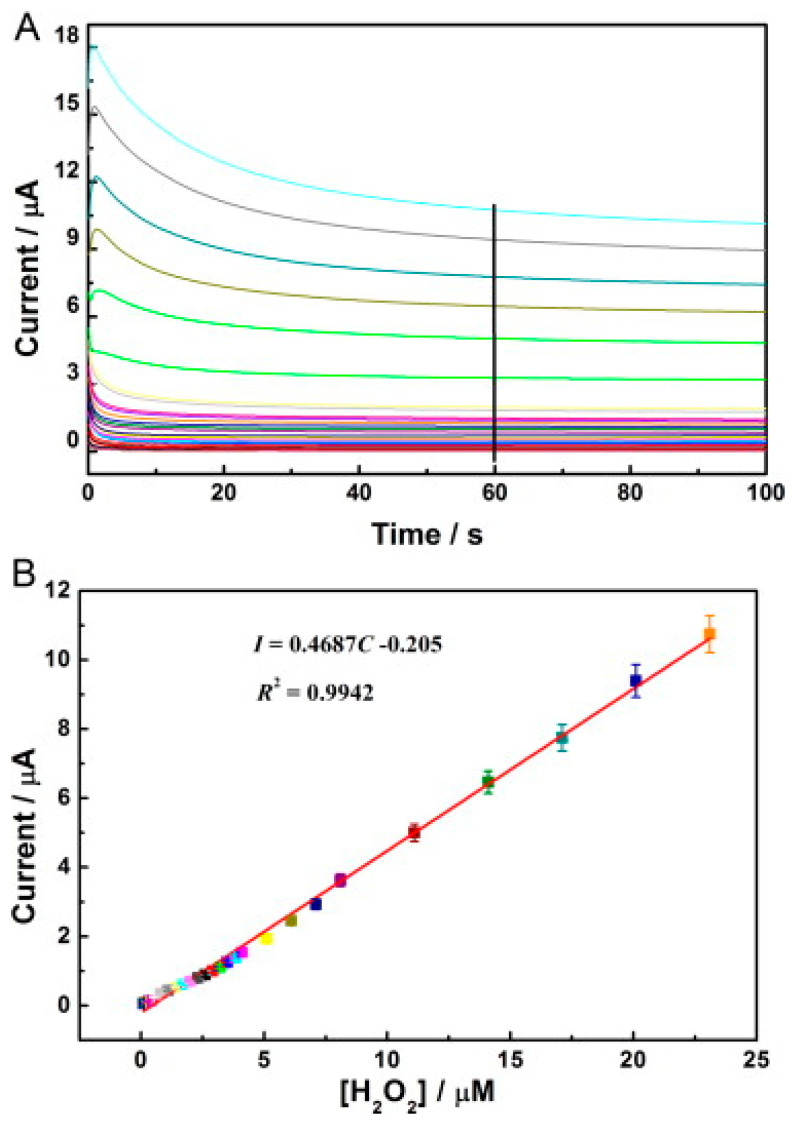
(**A**) Amperometric response of the HRP/C-Dots/LDHs/GCE to diverse concentrations of H_2_O_2_ from 0.1–23 µM in 0.1 M pH 7.0 PBS. The potential employed was −0.35 V. (**B**) The equivalent calibration curve of I-C acquired by chronoamperometry. Adapted from Ref. [93] with authorisation from Elsevier.

Another study demonstrating the immobilisation of horseradish peroxidase (HRP) on carbon nanodots/CoFe-layered double hydroxide (LDH) composites for the electrochemical detection of hydrogen peroxide was reported [93]. Carbon nanodots (C-Dots) were prepared as previously reported [94], while the preparation of C-dots/LDHs involve mixing C-Dots and CoFe-LDHs; after which, HRP was introduced to the mixture suspension of C-Dots/LDHs coupled with the addition of a known amount of Nafion to the resulting mixture for the stability of the modified electrode. The resulting mixture was finally drop cast on the previously treated GCE surface and dried up for 6 h at room temperature to obtain the HRP/C-Dots/LDHs/GCE. The electrochemical behaviour of HRP/C-Dots/LDHs GCE using cyclic voltammetry showed that C-Dots could enhance reaction involving the transfer of electron of HRP, despite HRP capacity to realise direct electrochemistry once immobilised by CoFe-LDHs (Figure 5). Overall, the electrocatalytic performance studies via cyclic voltammetry and chronoamperometry (summary in Table 1) revealed that the introduction of HRP boosted the responsiveness of HRP/C-Dots/LDHs/GCE, and as such was found to be better than C-Dots/LDHs/GCE owing to the merit of the material mixture in the design of electrochemical biosensors. Further, the outstanding analytical performance of HRP/C-Dots/LDHs/GCE toward H_2_O_2_ detection (Figure 6a,b) was attributed to the combined effect of HRP, C-Dots and CoFe-LDHs. 

Recently, N-rich carbon nanodots have been used by Tamara and his colleagues to develop a superb electochemiluminescence immunosensor for the detection of SARS-CoV-2 spike S1 protein [99]. The CNDTs provided the functional groups that were covalently attached to the SARS-COv-2 spike S1 antibody and improved the electrochemiluminescent signal in the Ruthenium redox probe. Over a broad concentration range of 2.5 to 240 pg/mL with a detection limit of 1.2 pg/mL, the immunosensor was highly selective towards the analyte. It is interesting to note that the immunosensor was utilised to detect the analyte in wastewater from rivers and cities.

**Figure 7:**
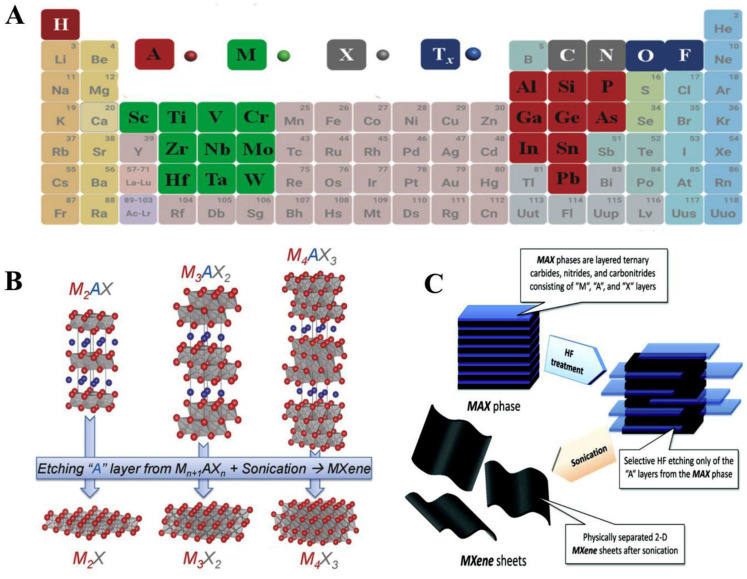
(**A**) A periodic classification demonstrating elements utilised for the MAX phases development (**B**) MAX phases employed for the synthesis of various MXenes (**C**) Synthesis of MXene sheets through exfoliation method. Reproduced from Ref. [95] with permission from Elsevier.

MXenes were first discovered and developed by the nanomaterials group, led by Prof. Yuri Gogotsi, collaboratively with Prof. Barsoum’s group, in 2011 at Drexel University, Philadelphia, USA. MXenes represent another family of two-dimensional (2D) transition metal carbides, carbonitrides and nitrides, relevant for numerous applications like electronics, energy storage and catalysis [100]. These layered materials are called ‘MXenes’ because they are synthesised by selectively etching A from the parent compounds, MAX phases. Out of the numerous etching approaches devised to synthesise MXenes, one of them is implemented by immersion of a MAX phase in hydrofluoric acid at room temperature. The suffix ‘ene’ in MXenes highlights their resemblance to graphene. MAX phases represent a group of over 60 members of layered ternary carbides and nitrides with a common formula M_n+1_AX_n_, where n = 1, 2, or 3, M is an early transition metal (like Sc, Ti, V, Cr, Nb), A represents an A-group element (mostly groups 13 and 14—Al, Si and Sn) and X is C and/or N [95,97]. MAX phase denotes a closely packed number of layered structures from alternating layers of M and A with a strong metallic-covalent M–X bond and weak M–A bond (Figure 7). MAX phases have good electrical and thermal conducting features, resistant to chemical shocks in addition to low thermal expansion coefficient [95,98]. Notably, MXenes have wide applications in energy storage, electromagnetic shielding/absorption, water purification, polymer nanocomposite fillers, electronic devices and optical conductive coatings [96]. The study of MXenes in electronic and optoelectronic applications, which is subsequently termed MXetronics is well documented [99,100,101]. Although there are numerous applications of MXenes in electronics, it is still less exploited for biological applications.

This section aims to highlight some of the existing few reports on MXene-based biosensors. The exploitation of MXenes in biosensing platforms has been recognised owing to their high metallic conductivity, biocompatibility, wide surface area, good ion-transmission features and ease of functionalisation [97,102,103]. Other exceptional merits of MXene materials, in contrast to other 2D nanomaterials, include a wide surface area with rich surface functional units like -O, -OH and -F, hence they are easily controlled to absorb biomaterials as well as modify the conducting properties, thereby enabling them to be used in sensing and biosensing [96,104,105,106,107]. Furthermore, the semi-conducting metallic features coupled with a suitable bandgap make MXenes to demonstrate low power loss in an off-state in contrast to graphene, and this helps to realise improved detection sensitivity [108]. MXenes began to gain recognition in biosensing applications after its exploration by Xu and co-workers in 2015 [108]. In this report, the authors employed a Ti_3_C_2_-MXene to fabricate an MXene-FET biosensor device for the detection of dopamine including investigating its neural activity. The MXene-FET biosensor device was able to practically detect dopamine at the lowest possible concentration of 100 × 10^−9^ M. According to the authors, this result was found to be lower than many already published works on graphene-based biosensors [109,110,111].

**Figure 8:**
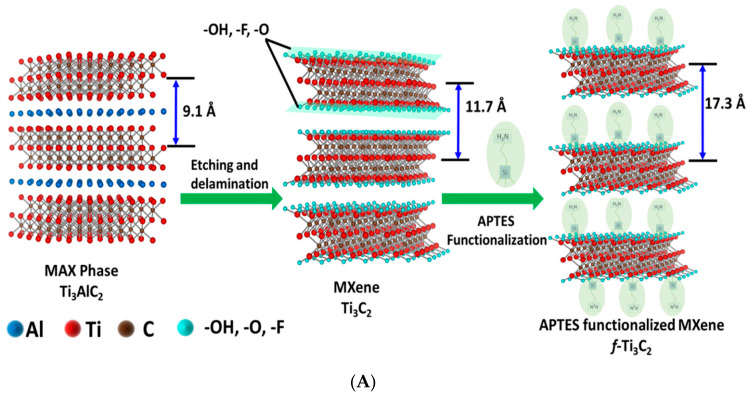

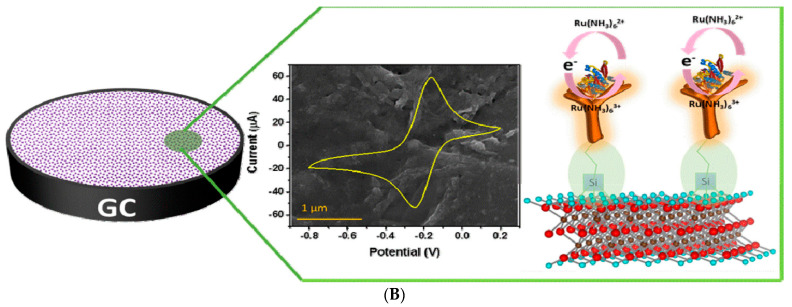
(**A**) Graphic model of Ti_3_C_2_-MXene functionalisation. Aluminum layer was etched from the Ti_3_AlC_2_-MAX phase, which delaminated into 2D nanosheets; the sheets composed of two layers of carbon intercalated within three tiers of titanium with the titanium surface arbitrarily surrounded with –OH, -O, and –F functional units. The Ti_3_C_2_-MXene surface was functionalised with APTES. Adapted from Ref. [112] with authorisation from Elsevier. (**B**) Design of the electrochemical CEA detection mechanism. Adapted from Ref. [112] with authorization from Elsevier.

For cancer biomarker detection, antibodies have been exploited to covalently bind to the amino units chemically initiated to the surface of a single/multilayer MXene (Ti_3_C_2_). This is due to the presence of densely populated functional units on the ultrathin 2D nanosheet of single/few layered Ti_3_C_2_-MXene, which mainly provides enhanced biomolecule loading and rapid access to the analyte [96,112]. Kumar et al. [112] designed a label-free biosensor with reference to ultrathin 2D Ti_3_C_2_-MXene nanosheets for CEA detection. The Ti_3_C_2_-MXene nanosheets for the biosensor design were prepared by minimally intensive layer delamination (MILD) technique [113], followed by an even functionalisation with 3-Aminopropyltriethoxysilane (APTES) for the covalent immobilisation of anti-CEA (Figure 8A). Cyclic voltammetry investigation of the redox probes impact on the electrochemical behaviour of the functionalised Ti_3_C_2_-MXene layers revealed hexaammineruthenium ([Ru(NH_3_)_6_]^3+^) as the preferred redox probe for biosensing. The choice of this redox probe was influenced by a sharp decline in oxidation current density of the ferrocyanide [Fe(CN)_6_]^−3/−4^ redox probe after the first cycle of the experiment. This was reported to be as a result of oxidation of Ti_3_C_2_-MXene layers at higher potentials (−0.1 V to 0.8 V), which led to the production of TiO_2_ over the MXene sheets [107]. Contrary to that observation, [Ru(NH_3_)_6_]^3+^ redox probe was oxidised and reduced at lower potential windows (Figure 8B). Additionally, covalent immobilisation of the bioreceptor resulted in greater uniformity, distribution and a densely populated bonded biomarker. Overall, the biofunctionalised MXene-based device displayed an observed detection limit of 0.000018 ng mL^−1^ over a very broad range of 0.0001–2000 ng mL^−1^ and sensitivity of ~37.9 µA ng^−1^ mL cm^−2^ per decade for CEA [112]. 

**Figure 9:**
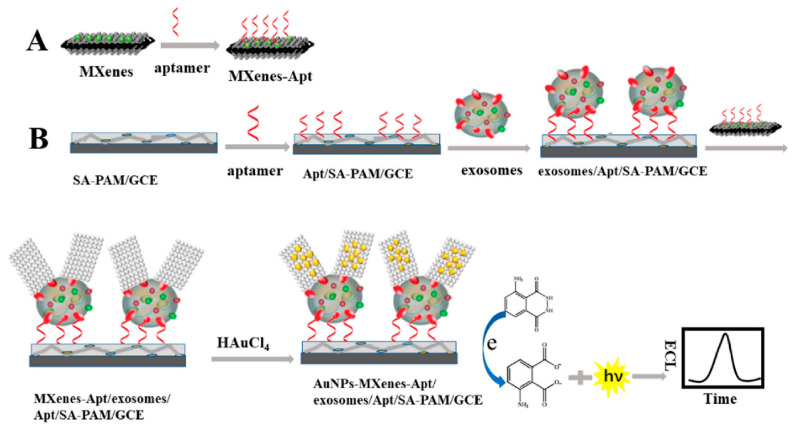
Principle of the ECL biosensor for the detection of exosomes with respect to in situ formation of AuNPs decorated Ti_3_C_2_ MXenes nanoprobes. Adapted from Ref. [114] with authorisation of ©2020 American Chemical Society.

The large surface area of MXenes has been reported to provide an outstanding conducting aid to accept additional AuNPs and recognition sites. As a result, the development of an extremely sensitive electrogenerated chemilumiscence (ECL) biosensor via in situ formation of AuNPs decorated Ti_3_C_2_ MXenes hybrid with modified aptamer (AuNPs-MXenes-Apt) for exosomes detection was described [114]. In this study, the authors employed exosomes recognised CD63 aptamer, modified on sodium alginate (SA) and poly (acrylamide) (PAM) modified electrode to efficiently capture the exosomes on the SA-PAM electrode interface. Firstly, the in situ formation of AuNPs-MXenes-Apt hybrid aided the active identification of exosomes. Secondly, it provided a bare catalytic surface with significant electrocatalytic activity of AuNPs. The synergistic catalytic influence of the AuNPs-MXenes-Apt on the ECL reaction to luminol resulted in exosomes detection limit of 30 particles µL^−1^ over a range of 10^2^–10^5^ particles µL^−1^. The authors claimed that the observed LOD derived from HeLa cell line in this study is 1000 times way less than the established ELISA technique. Figure 9A,B show the scheme for the fabrication of an ECL biosensor for detecting exosomes. The GCE surface modified the SA-PAM layer, which provided the carboxyl group for CD63 Apt immobilization, resulting in more CD63 Apt molecules modified on the electrode. Based on the high specificity of Apt and CD63 protein on the surface of exosomes, the electrode was incubated in the MXenes-Apt solution after exosomes were captured.

Researchers have also found that the bioactivity of lively protein in a biosensor typically drops upon direct contact with the electrode surface. Therefore, some materials are carefully chosen to immobilise lively proteins and preserve their action. In this regard, MXenes have been found useful as protein immobilisation platform because they help to protect the lively proteins and aid the immediate transfer of electron between the enzyme and the electrode, resulting in the possibility of devising mediator-free biosensors [115]. On this note, Ti_3_C_2_T_x_ was selected for immobilisation of hemoglobin (Hb) on a Nafion/Hb/Ti_3_C_2_T_x_/GCE biosensor for nitrites detection. Owing to the reasonably good conductivity of Ti_3_C_2_T_x_, the Nafion/Hb/Ti_3_C_2_T_x_/GCE biosensor demonstrated a high-impact operation with a magnified detection range of 0.5–11800 µM and impressive LOD of 0.12 µM [104]. 

**Figure 10:**
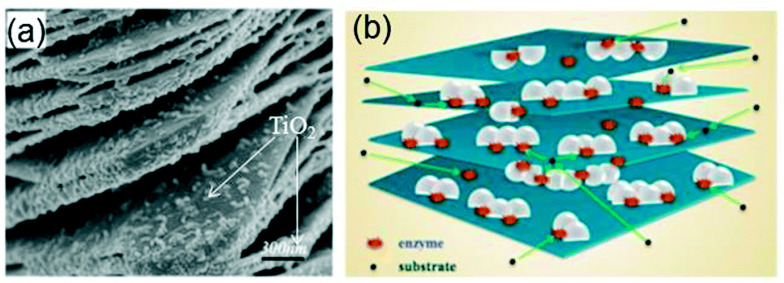
(**a**) SEM image of the TiO_2_-Ti_3_C_2_ nanocomposite. (**b**) Graphics of the TiO_2_-Ti_3_C_2_ nanocomposite. Reproduced with authorisation from Refs. [105,115]. Copyright of Elsevier and Royal Society of Chemistry (RSC).

A TiO_2_-modified Ti_3_C_2_T_x_ nanocomposite, prepared using hydrothermal synthesis, was utilised as an immobilisation medium for hemoglobin (Hb) in the fabrication of a Nafion/Hb/Ti_3_C_2_T_x_-TiO_2_/GCE biosensor for H_2_O_2_ detection [105]. The SEM image showed that the microstructure of the nanocomposite of Ti_3_C_2_T_x_ nano-layers (Figure 10a) was composed of an organ-like arrangement with a closed and opened edge, coupled with the many white TiO_2_ nanoparticles loaded on the Ti_3_C_2_T_x_ layers. The organ-like arrangement was found to be beneficial for enzyme immobilisation on the inner surfaces of Ti_3_C_2_T_x_. Furthermore, the outstanding biocompatibility and chemical stability of TiO_2_ provided a protective micro-environment for the enzymes. Figure 10b depicts the graphics of the TiO_2_-Ti_3_C_2_T_x_ nanocomposite capturing hemoglobin. The enzyme was immobilised on the internal sides of the organ-like designed Ti_3_C_2_T_x_ nano-layers adjacent to TiO_2_, thus ensuring the strength and activity of the enzyme much longer. Overall, the Nafion/Hb/Ti_3_C_2_T_x_-TiO_2_/GCE displayed a faster response time below 3 s in addition to a broader linear range of 0.1–380 µM in contrast to Nafion/Hb/Ti_3_C_2_T_x_/GCE where Ti_3_C_2_T_x_ was utilised as the immobilisation medium [105,106]. 

The summary of the application of carbon-based nanocomposite is highlighted in Table 2.

Another class of carbon nanomaterial is graphitic carbon nitrides (GCN); they are a remarkable class of conjugated polymer semiconductors and have fascinated the attention of scientists owing to their unique analytical merits including biocompatibility, avalanche nitrogen lone-pairs, surface defects, extended π-electron density and presence of intrinsic peroxidase activity, which is enhanced when attached to biomolecules [115,116,117]. These interesting features have endorsed the application of GCN in the development of biosensors. For instance, Walaa and his colleagues developed a brilliant porous GCN in collaboration with gold nanoparticles for the detection of cardiac Troponin I [118]. The author affirmed that the modification of the electrode surface with GCN had a significant impact on the interfacial electron transport, aptamer immobilization, and active surface area, and improved the performance of the biosensor. Interestingly, the fabricated aptasensor was integrated into a miniaturized potentiostat and wirelessly connected to a smartphone, which affirms its potential for point-of-care diagnostics application. Similarly, mesoporous-C_3_N_4_ was encapsulated with AuNPs (mpg-C_3_N_4_@AuNPs) and used for the development of surface-enhanced Raman spectroscopy substrate for the detection of 6-thioguanine (6-TG) [119]. Interestingly, the agglomeration of gold nanoparticles was successfully prevented because the gold nanoparticle grew inside the nanopores and on the surface of the mesoporous-C_3_N_4_. The author submitted that the hybrid of mpg-C_3_N_4_@AuNPs was beneficial to SERS enhancement because of the intrinsic properties of both modifiers coupled with the π–π affinity between GCN and the analyte. The platform was able to detect a wide linear range of the analyte from 0.6 μM to 0.48 μM with a detection limit of 0.24 μM.

A one-pot synthesis of novel ternary nanocomposite of ceria, polypyrrole (Ppy), and graphitic carbon nitride (CeO_2_/Ppy@g-C_3_N_4_) was used for the development of a non-enzymatic biosensor for the detection of quinol in water [120]. The electrochemically active surface area of the modified electrode was 2.3 increment in comparison with the bare electrode, and the platform was able to detect a broad concentration range of quinol from 0.01 to 260 μM and the detection limit of 1.5 nM was obtained. It was highlighted that Ppy was used because it is highly conductive, stable, and its synthesis protocols involve simple preparation steps. However, the author was unable to compare the individual conductivity of the material used for the sensor development because of the one-pot synthesis used in the preparation of the nanocomposite (CeO_2_/Ppy@g-C_3_ N_4_).

Yujuan and his team developed a sensor for the detection of acetaminophen (AP) by GCN and EDOT (3,4-ethylene dioxythiophene) [121]. The GCN@EDOT was electrochemically co-deposited on the GCE by oxidizing and polymerisation of EDOT. The co-electrodeposition was done by cycling a potential from 0.2 to 1.2 V for ten cycles at a scan rate of 100 mV/s using cyclic voltammetry. The author noted that the co-detection of GCN and EDOT was due to π–π interaction, and the platform was used in the detection of AP in human serum. The platform was reported to be reproducible, stable, and selective. In our opinion, the stability of the sensor is a result of the electrodeposition route used in the sensor fabrication.

Nanocellusose (NC) is another type of polymer that has fascinated the interest of researchers; it is made from cellulose using mechanical or chemical techniques. Grinding and high-pressure homogenization are used in the mechanical preparation of nanocellulose to create cellulose nanofibrils with 500–2000 nm length and 5–50 nm diameter [122,123,124]. While the chemical process encompasses delignification, alkali treatment, mechanical breakdown, and acid hydrolysis, the result is rod-like cellulose nanocrystals with diameters and lengths of 3–50 nm and 100–500 nm, respectively [122,123,124]. NC has a great surface area, good cytocompatibility, low toxicity, biodegradable, biocompatible, and low density [125,126]. These analytical merits have drawn attention to the use of NC in biosensor development. For example, NC was used in collaboration with single-wall carbon nanohorns to detect adenine and guanine bases in RNA and DNA. The platform was said to have strong catalytic activity and antifouling properties, and it detected guanine to a limit of 1.4 ×10^−6^ M [127].

An electrochemical biosensor was developed from peptide nuclide acid (PNA)@reduced graphene oxide (NH_2_-rGO/2,2,6,6-tetramethylpiperidin-1-yl)oxyl nanocrystalline cellulose (TEMPO-NCC) and used for the detection of Mycobacterium tuberculosis (M. Tuberculosis) [128]. The nanohybrid films (rGO-TEMPO-NCC) were drop-coated on a screen-printed carbon electrode (SPCE). The author affirmed that the sensor was able to differentiate between complementary, non-complementary and one-base mismatch DNA sequences using methylene blue (MB) as the electrochemical indicator. The fabricated biosensor detected the analyte with a linear calibration curve in a concentration range of 1 × 10^−8^–1 × 10^−13^ and a detection limit of 3.14 × 10^−14^ M was reported.

Solin and co-workers employed EDC/NHS coupling chemistry with nanocellulose to create efficient anchor layers for the immobilization of anti-immune complex antibodies on paper-based immunoassay for the detection of tetrahydrocannabinol [129]. The authors used the OH-group-rich surface, high surface-to-volume ratio, and high hygroscopicity of tempo-oxidized cellulose nanofibrils (TOCNF) to enhance effective surface functionalisation and promote water permeation inside the nanocellulose network structure, providing a hydrophilic spacer for sensing antibodies. The conjugation of the probe onto the electrode surface was carried out by incorporating the thiol group at the N-terminal of the PNA. 

## 2. Conclusions, Recommendation and Future Perspective

In conclusion, this review has clearly identified and demonstrated the special capabilities of carbon nanomaterials and new member of 2D materials, MXenes, in biosensor development. Hence, they are considered superb and promising immobilisation candidates for the development of high-impact biosensors. Carbon nanomaterials possess many remarkable features, some of which include broad potential window, chemical inertness, cost effectiveness, enhanced surface area, electrical and thermal conductivity, superior biocompatibility and photophysical attributes. MXenes, at the same time, have high metallic conductivity, biocompatibility, good ion-transmission characteristics, ease of functionalisation, and wide surface area with rich surface functional units. The topic also apparently found out that modification of the functional units of nanomaterials and integrating them with other materials with excellent characteristics usually boost device performance, sensitivity, selectivity, shelf-life, and detection limits. 

We firmly recommend that researchers should adopt environmentally friendly synthesis techniques and investigate the chemistry of interaction between the nanocomposites utilised in the development of biosensors. Additionally, at least two electrochemical techniques must be utilised for characterising the modifiers utilised and detecting various analytes. Furthermore, the electrodeposition approach should be used to immobilise the modifiers on the conducting substrates; however, if this is not done, we advise using nafion as a stabilising agent for the modifiers.

For future research, we recommend primarily that more innovative and effective surface modifications are needed for both carbon nanomaterials and MXenes to be able to satisfy the requirements of various biomedical applications. Robust but designated modification methods are envisaged to accomplish a more robust and proper detection. Based on the above considerations, there is a pressing need for an in-depth study on carbon and MXenes-based materials in addition to their composites. It is also of great importance to take note properly that only inter-disciplinary collaborative responsibility can result into cutting-edge research. Such a perspective is very much needed in the world of science in all corners of the globe; hence, the innovative discovery and in-depth understanding of the developed nanomaterials will disclose greater significant uses. The key objective will aim at final clinical and commercial use of carbon and MXene-based biosensing devices.

## Figures and Tables

**Table 1:** Comparing the analytical performances of HRP/C-Dots/LDHs/GCE with other biosensors.

Electrode Material	Linear Range (µM)	LOD (µM)	Sensitivity (mA mM^−1^ cm^−1^)	Reference
C-Dots/GCE	1.0–3.5	0.55	0.055	[93]
LDHs/GCE	1.0–6.0	0.68	0.061	[93]
C-Dots/LDHs/GCE	0.5–7.5	0.46	0.12	[93]
HRP/C-Dots/LDHs/GCE	0.1–23.1	0.04	0.47	[93]
HRP-Ag@C/ITO	0.5–140	0.2	-	[95]
HRP/RTIL/GNPs-TNTs/Nafion	5.0–1000	2.1	-	[96]
Nafion/HRP/Zr-IP6/GCE	0.667–6.0	0.53	-	[97]
Gold-nanoparticle-adsorbed poly (thionine) film	5–150	1.5	-	[98]

**Table 2:** Reveals the summary of various carbon nanomaterials used in the development of biosensors for various analytes.

Electrodes	Linear Range (ng/mL)	Detection Limit (ng/mL)	Analyte	Reference
rGO/Pt@Zn-Mof-74	0.6–600	0.18	Glucose	[22]
rGO@PANI	0.1–1000	0.00301	BRCA1	[28]
CoTAPC-rGO@CdsNPs	0.001–5	0.00063	PSA	[43]
CMC@rGO	20–500	0.2	Catechol	[46]
CNTs@PANI	1–10	0.33	MTB	[54]
GoX-CNT	0.25–499	0.003677	Glucose	[55]
cMWCNTs@PANI	5–100	0.02900	Paracetamol	[56]
AuNPs@CNPs	0.005–100	0.0019	AFP	[70]
AuNPs@CNPs	0.5–100	0.0092	As(III)	[72]
CNPs	0.5–100	0.032	microRNA	[75]
PPI@CNDTs	0.005–300	0.00145	CEA	[78]
Polyamidoamine@CNDTs	1–80	0.33	AFP	[90]
Nafion@CNDTs	10–320	0.001	AFP	[92]
HRP@CNDTs@CoFe	1–23.1	0.04	H_2_O_2_	[93]
*f*-Ti_3_C_2_-MXene	0.1–200	0.00379	CEA	[112]
*f*-Ti_3_C_2_-MXene@AuNPs	0.001–1000	0.00333	Exosomes	[114]

## Data Availability

Not applicable.
